# Bioactivity Screening and Genomic Analysis Reveals Deep-Sea Fish Microbiome Isolates as Sources of Novel Antimicrobials

**DOI:** 10.3390/md21080444

**Published:** 2023-08-07

**Authors:** Shona Uniacke-Lowe, Fergus W. J. Collins, Colin Hill, R. Paul Ross

**Affiliations:** 1 Department of Microbiology, University College Cork, T12 K8AF Cork, Irelandc.hill@ucc.ie (C.H.); 2 APC Microbiome Ireland, T12 K8AF Cork, Ireland; 3 Teagasc Food Research Centre, P61 C996 Fermoy, Ireland

**Keywords:** deep-sea, bacteriocins, antimicrobial, biosynthetic gene clusters, genome mining, fish microbiome, antimicrobial resistance

## Abstract

With the increase in antimicrobial resistance and the subsequent demand for novel therapeutics, the deep-sea fish microbiome can be a relatively untapped source of antimicrobials, including bacteriocins. Previously, bacterial isolates were recovered from the gut of deep-sea fish sampled from the Atlantic Ocean.In this study, we used in vitro methods to screen a subset of these isolates for antimicrobial activity, and subsequently mined genomic DNA from isolates of interest for bacteriocin and other antimicrobial metabolite genes. We observed antimicrobial activity against foodborne pathogens, including *Staphylococcus aureus*, *Listeria monocytogenes*, *Enterococcus faecalis* and *Micrococcus luteus*. In total, 147 candidate biosynthetic gene clusters were identified in the genomic sequences, including 35 bacteriocin/RiPP-like clusters. Other bioactive metabolite genes detected included non-ribosomal peptide synthases (NRPS), polyketide synthases (PKS; Types 1 and 3), beta-lactones and terpenes. Moreover, four unique bacteriocin gene clusters were annotated and shown to encode novel peptides: a class IIc bacteriocin, two class IId bacteriocins and a class I lanthipeptide (LanM subgroup). Our dual in vitro and in silico approach allowed for a more comprehensive understanding of the bacteriocinogenic potential of these deep-sea isolates and an insight into the antimicrobial molecules that they may produce.

## 1. Introduction

The deep sea is one of the largest biomes on Earth, encompassing 95% of the Earth’s oceanic volume and reaching depths of over 10,000 m [1,2]. It is a unique environment that is characterised by low temperatures, high hydrostatic pressure—which increases by one atmosphere with every 10 m increase in water depth—low oxygen, and no sunlight beyond 1000 m [3]. These conditions are relatively constant, with little impact from ocean currents and seasonality [4]. However, it is estimated that less than 0.001% of the deep sea has been explored, and relatively little is known about its microbial inhabitants compared to those of the terrestrial world [2]. The deep sea is, perhaps, one of the last frontiers of biodiscovery and, as such, has been gaining attention as a source of potential novel microbial compounds.

Deep-sea microorganisms are uniquely adapted to the extreme conditions of their environmental niche. Notably, deep-sea microbial proteins are often optimally active at low temperatures [5] and can have unusual amino acid structures [6], leading to an increased tolerance to high pressure [7,8].

Some recently described antimicrobials are produced by novel deep-sea bacteria isolated from deep-sea sediment. These include marthiapeptide A, a cyclic peptide produced by *Marinactinospora thermotolerans* [9]; lobophorins E, F and K, antibiotics from *Streptomyces* spp. [10,11]; and anthracimycin, an anti-anthrax (*Bacillus anthracis*) antibiotic produced by *Streptomyces* sp. CNH365 [12]. Other such antimicrobials have also been found in deep seawater, for example, brainimycins B and C (macrolides) produced by *Pseudonocardia carboxydivorans* [13]. Deep-sea bacteria found in association with higher organisms are also now gaining attention as producers of novel antimicrobials, particularly those from deep-sea coral [11] and sponges [14].

Bacteriocins are small (<10 kDa), ribosomally synthesised antimicrobial peptides produced by bacteria that target other bacteria, and to which the producer has immunity. Bacteriocins can be divided into two main classes: class I (lantibiotics or lanthipeptides) and class II (non-lanthionine-containing bacteriocins) [15]. Lanthipeptides can be further subdivided into five subclasses, I–V, based on the biosynthetic enzyme(s) involved in their modification [16]. The class II bacteriocins are subdivided into four groups: the anti-listerial pediocin-like peptides (class IIa), two-component peptides (class IIb), cyclic/circular bacteriocins (class IIc) and the linear and non-pediocin-like peptides (class IId) [17]. Bacteriocins are emerging as alternatives to antibiotics due to their spectrum of activity—which can be broad or narrow—heat stability and their capacity for bioengineering to generate derivatives with value-added properties [18]. This is of particular importance, given the significant and growing threat that antibiotic-resistant organisms pose in the medical and food industries. According to a 2019 report by the Centres for Disease Control and Prevention (CDC), almost three million infections, including foodborne illness, caused by antibiotic-resistant organisms occur each year in the United States of America alone [19].

To date, very few studies have explored the production of bacteriocins from deep-sea fish microbial isolates. One example is BaCf3, a bacteriocin produced by *Bacillus amyloliquefaciens* BTSS3, isolated from the intestine of a deep-sea shark. This highly thermostable bacteriocin was initially identified through in vitro screening methods and showed inhibitory activity against pathogenic bacteria, including *Bacillus cereus*, *Clostridium perfringens*, *Staphylococcus aureus* and *Salmonella enterica* Typhimurium [20,21].

Previous work carried out in our laboratory generated metagenome-assembled genomes (MAGs) from shotgun metagenomic sequencing of intestinal microbiomes samples from deep-sea fish from the Atlantic Ocean [22]. The fish were collected as part of a groundfish survey. Analysis of the MAGS revealed information on the taxonomic and functional diversity of these samples, such as a predominance of Pseudomonadota (Proteobacteria), as well as a large abundance of genes involved in DNA repair, protein folding and motility. Bacterial isolates were recovered from the intestines and skin of a subset of these deep-sea fish and were preliminary screened for bioactivity against the Gram-positive target organism, *Lactobacillus delbrueckii* subsp. *bulgaricus*. *L. bulgaricus* is commonly used in the assessment of antimicrobial activity due to its low-pH resistance and sensitivity to a wide range of bacteriocins [23,24,25]. In this study, a cohort of these bacterial isolates were reassessed for bioactivity against the target strain, *Lactobacillus delbrueckii* subsp. *bulgaricus,* and were subsequently screened against a range of various target strains.

The aim of this study was to determine the bacteriocinogenic (ability to produce bacteriocins) potential of deep-sea fish microbial isolates (n = 65) through in vitro screening against various target strains, including pathogenic bacteria, and through in silico mining of the genomes of a selection of these isolates (n = 36) for putative bacteriocin biosynthetic gene clusters. We also looked for the presence of other secondary metabolite gene clusters and antimicrobial resistance (AMR) genes.

## 2. Results

**Figure 1:**
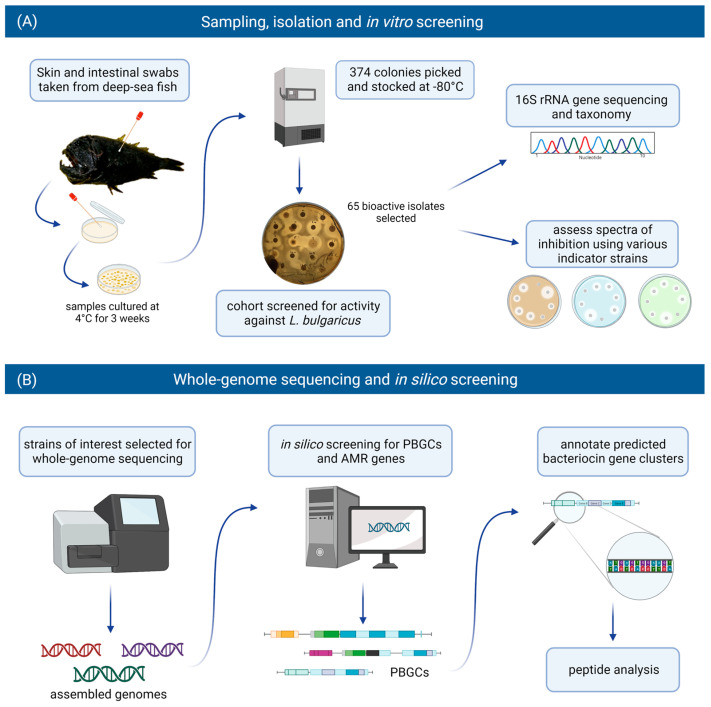
The in vitro and in silico-based approaches used in this study to assess the bacteriocinogenic potential in the deep-sea fish microbiome. (**A**) Swabs were taken from the skin and intestines of deep-sea fish. Samples were cultured at 4 °C for three weeks, and 374 isolates were recovered. A cohort of these isolates were screened for activity against *Lactobacillus delbrueckii* subsp. *bulgaricus*. Sixty-five bioactive isolates were identified and selected for further in vitro screening and identification through 16S rRNA gene analysis. (**B**) Strains of interest were selected for whole-genome sequencing and subsequent in silico screening for bacteriocin/RiPP genes, putative biosynthetic gene clusters (PBGCs) and antimicrobial resistance (AMR) genes. Predicted bacteriocin gene clusters were annotated, and the bacteriocin peptide precursors were analysed (figure created with BioRender.com).

For this study, we included bacteria that had been isolated from the skin and intestines of deep-sea fish and assessed them for antimicrobial activity against various target strains, including foodborne pathogenic bacteria. Those producing antimicrobial activity were identified by 16S rRNA gene analysis. Isolates of interest were selected for whole genome sequencing and subsequent in silico screening for putative bacteriocin gene clusters and putative biosynthetic gene clusters (PBGCs) encoding secondary metabolites. Strains were also assessed for the presence of antimicrobial resistance (AMR) genes. The workflow implemented in this study is illustrated in Figure 1.

### 2.1. Recovery and Selection of Bioactive Isolates

**Figure 2:**
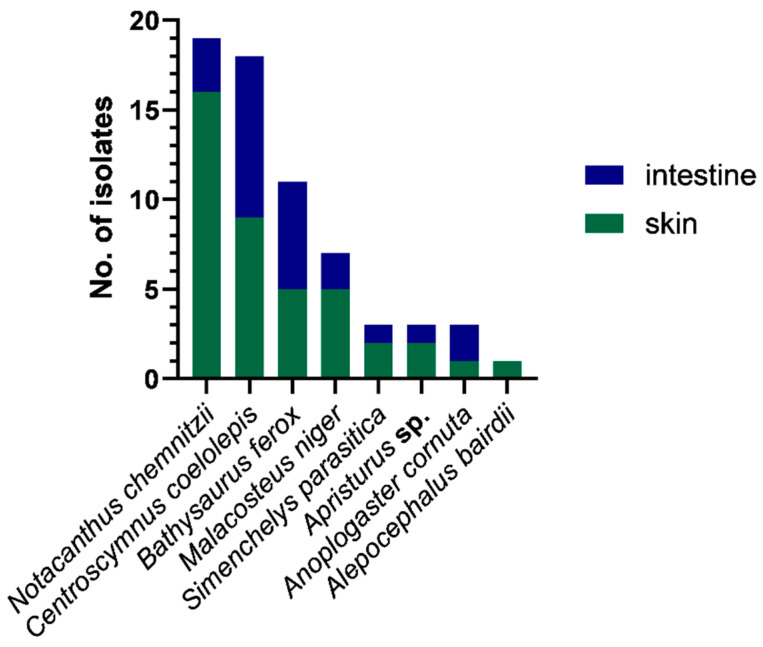
Total counts of isolates recovered per sample type (skin or intestine swab) and per fish host species. The majority of isolates were from skin swabs of *Notacanthus chemnitzii* and *Centroscymnus coelolepis.*

The 65 bacterial isolates came from eight different deep-sea fish, namely: *Alepocephalus bairdii*, *Anoplogaster cornuta*, *Apristurus* sp., *Bathysaurus ferox*, *Centroscymnus coelolepis*, *Malacosteus niger*, *Notacanthus chemnitzii*, and *Simenchelys parasitica*. The majority (63%) of the isolates came from skin swabs, with most isolates recovered from the deep-sea fish *N. chemnitzii* and *C. coelolepis* (Figure 2).

We confirmed that 44 of the 65 isolates were active against *L. bulgaricus* (Table 1). All the isolates grew on marine agar (MA) with minimal colony swarming, though modified tryptic soy agar (mTSA) resulted in slightly greater inhibitory activity. Twenty-one and eighteen of the isolates did not grow on brain heart infusion (BHI) or mTSA, respectively, under these conditions. In instances where inhibition could not be measured because of colony swarming or poor growth of the indicator, the result was recorded as “no data” (nd; Table 1).

### 2.2. Taxonomic Diversity

**Figure 3:**
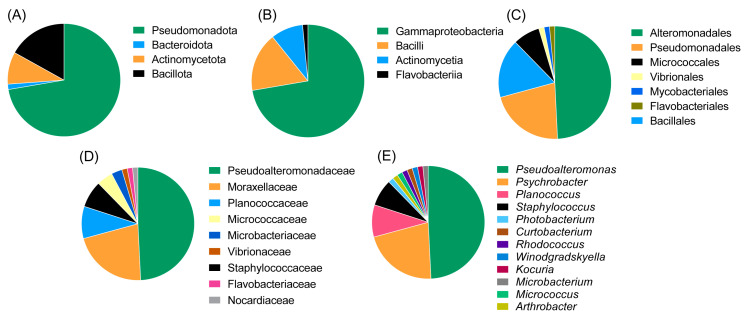
Diversity of the deep-sea fish microbiome isolates at the phylum level (**A**), class level (**B**), order level (**C**), family level (**D**) and genus level (**E**). The most abundant isolates in this study were *Pseudoalteromonas* and *Psychrobacter* species of the phylum Pseudomonadota (Proteobacteria).

The majority of the strains belonged to the class Gammaproteobacteria (of the phylum Pseudomonadota, at 72.3%) followed by Bacilli (phylum Bacillota 16.9%), Actinomycetia (phylum Actinomycetota, 9.2%) and Flavobacteriia (phylum Bacteroidota, 1.5%). The most abundant orders were Alteromonadales (49.2%), Pseudomonadales (20%) and Bacillales (16.9%). Other represented orders were Micrococcales, Mycobacteriales, Flavobacteriales and Vibrionales. The most abundant genus was *Pseudoalteromonas* (family *Pseudoalteromonadaceae*, 51.9%), followed by *Psychrobacter* (family *Moraxellaceae*, 21.5%), *Planococcus* (family *Planococcaceae*, 9.2%) and *Staphylococcus* (family *Staphylococcaceae*, 7.7%). The remaining genera were represented only by a single isolate; these included *Winogradskyella*, *Rhodococcus*, *Arthrobacter*, *Kocuria*, *Curtobacterium, Photobacterium* and *Micrococcus*. An overview of the taxonomic diversity of the isolates is shown in Figure 3.

### 2.3. Spectrum of Activity

**Figure 4:**
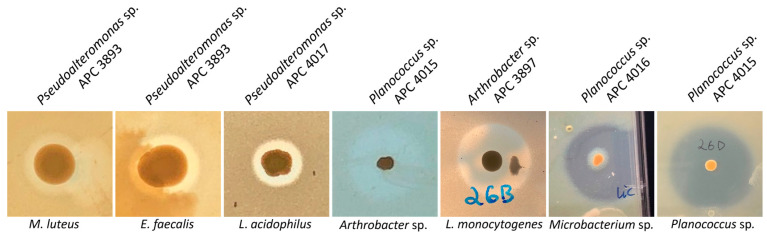
Representative zones of inhibition of the bioactive marine isolates. The strongest inhibition was demonstrated against other marine isolates, *Planococcus* sp. and *Microbacterium* sp. and *Listeria,* including *L. monocytogenes*. The producer IDs are given above the images, and the indicator strains are below.

**Figure 5:**
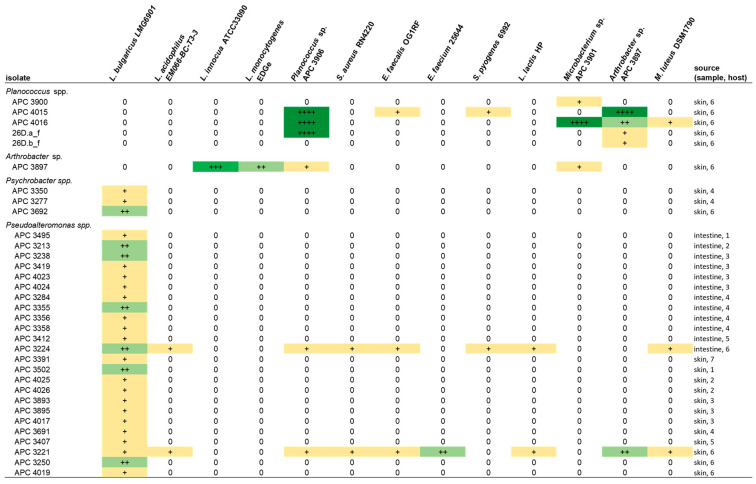
The spectrum of inhibition of the bioactive deep-sea isolates against the target strains by colony overlay assays on marine agar. The overall broadest range of activity was exhibited by *Pseudoalteromonas* strains APC 4017 and APC 3893. Inhibition is indicated by + (0.5–2.5 mm), ++ (2.6–5 mm) and +++ (5.1–10 mm) and is coloured to highlight inhibition patterns. Only the results of strains that demonstrated inhibitory activity under these conditions are reported in the table.

Following the initial screen against *L. bulgaricus*, the isolates were tested against a range of different target strains, including foodborne pathogens, food spoilage bacteria and marine bacteria. MA was chosen as the culture medium for the spectrum of activity screen as it supported the growth of the widest range of the marine isolates as compared to BHI and mTSA. Representative zones of inhibition are shown in Figure 4. Overall, 13 of the 22 target strains were inhibited (Figure 5). Four of the deep-sea strains that initially showed little or no activity against *L. bulgaricus* after culturing on marine agar were able to inhibit several of the other strains. Isolates of the genus *Planococcus* (strains APC 3900, APC 4015, APC 4016, 26D.a_f and 26D.b_f) were most effective at inhibiting the more closely related indicators: *Planococcus* sp. APC 3906 (of the same genus) and those of the order Micrococcales: *Arthrobacter* sp. APC 3897, *Microbacterium* sp. APC 3901 and *Micrococcus luteus*. *Planococcus* sp. APC 4016 additionally showed limited activity against *E. faecalis* and *S. pyogenes*. *Arthrobacter* sp. APC 3897 showed the greatest activity against *L. innocua* and *L. monocytogenes*. This isolate also showed activity against the *Planococcus* sp. and *Microbacterium* sp. indicators. *Pseudoalteromonas* sp. APC 4017 had the broadest spectrum of activity, inhibiting nine of the twenty-two indicators. None of the isolates inhibited *B. cereus*, *S. intermedius*, *Rhodococcus* sp. APC 3903 or *Kocuria* sp. APC 4018. None of the Gram-negative target strains (*E. coli*, *P. aeruginosa*, *S. Typhumurium*, *V. fischerii*, *Psychrobacter* sp. APC 3276) were inhibited. Thirteen of the isolates had no inhibitory effects against any of the indicators under these conditions. It was observed that several strains from the same sample site and the same host produced identical inhibition patterns and are likely to be identical strains. For example, *Psychrobacter* sp. strains APC 3350 and APC 3277, both from the skin of *Centroscymnus coelolepis,* only inhibited *L. bulgaricus*; similarly, *Pseudoalteromonas* sp. strains APC 4020 and APC 4023 from the intestine of deep-sea fish *Bathysaurus ferox* both also inhibited only *L. bulgaricus*.

### 2.4. In Silico Antimicrobial Screening

A selection of isolates that demonstrated in vitro antimicrobial activity and a representative of the identified taxonomic genera were chosen for whole genome sequencing. A total of 36 genomes were sequenced to enable genome-based in silico screening to identify antimicrobial-encoding genes and to identify potential bacteriocin producers that may not have shown in vitro activity under our tested conditions. The genome assemblies were cross-checked against the MAG sequence data (from [22]), and it was confirmed that the isolate sequences were not present in the MAGs.

**Figure 6:**
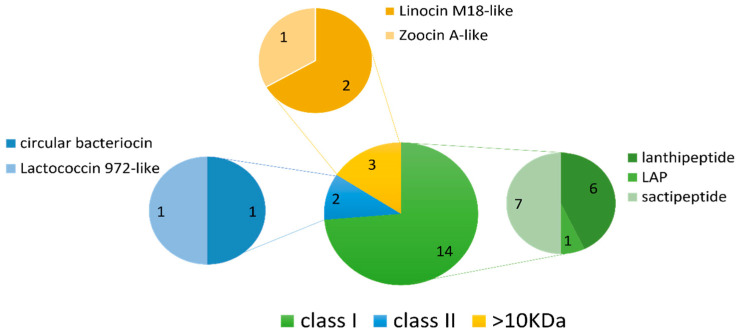
Summary of the bacteriocin classes and their subtypes identified using BAGEL4. Nineteen AOIs were predicted in total across the 36 deep-sea fish gut isolate genomes. Sactipeptides and lanthipeptides (class I bacteriocins) were the most frequently predicted bacteriocin subtypes.

The BAGEL4 mining software searches for potential motifs and core peptides of ribosomally synthesised and post-translationally modified peptides (RiPPs), such as bacteriocins, as well as larger antimicrobial peptides (>10 kDa), and presents each output as an area of interest (AOI) [26]. Nineteen AOIs were predicted by BAGEL4 across 13 of the 36 genomes consisting of fourteen class I bacteriocins, two class II bacteriocins and three larger antimicrobial proteins (>10 kDa) (Table 2 and Figure 6). Sactipeptides were the most abundant sub-type (seven hits), followed by lanthipeptides (six hits). Of the lanthipeptide hits, three were of the lanthipeptide subclass II, sharing 50–56% similarity to the bacteriocins mersacidin and cerecidin (Table 2). The remaining three lanthipeptides were subclass III; however, they lacked an identifiable core peptide gene within the predicted gene cluster. The remaining bacteriocin/RiPP sub-types included one linear-azol(in)e-containing peptide (LAP), one class II circular bacteriocin with similarity to circularin A and one class II bacteriocin with similarity to lactococcin 972. Four AOIs each belonged to *Planococcus* strains, *Pseudoalteromonas* strains and the *Rhodococcus* sp. strain APC 3903. The remaining AOIs belonged to *Arthrobacter* sp. APC 3897, *Curtobacterium* sp. APC 3903, *Kocuria* sp. APC 4018, *Psychrobacter* sp. APC 3350 and *Winogradskyella* sp. APC 3343.

**Figure 7:**
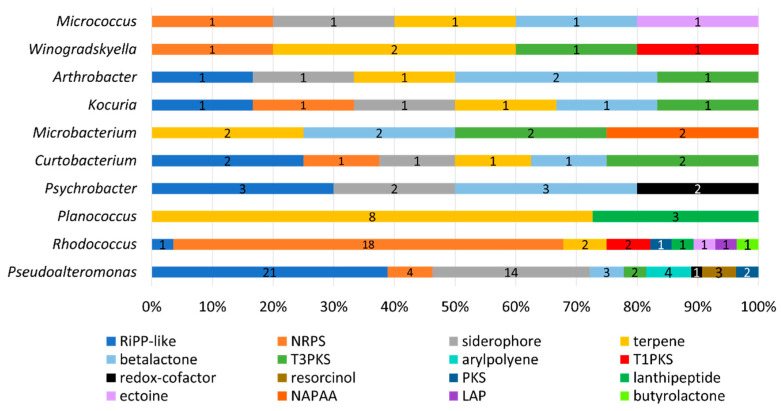
PBGC hits predicted by the AntiSMASH database of secondary metabolites and antibiotics. Results are given as a percentage of the total per genus, and counts of each PBGC type are given. Default search strictness parameters were used. The most frequent hits included RiPPs and lanthipeptides, which suggests these isolates encode bioactive compounds, such as bacteriocins. Other gene cluster hits include polyketide synthases, siderophores, terpenes and non-ribosomal peptide synthases (NRPS).

AntiSMASH identified 141 putative biosynthetic gene clusters (PBGCs) encoding secondary metabolites, with PBGCs being found in all 36 genomes (Table 3 and Figure 7). The most frequent PBGC types identified were RiPP-like (29 hits), non-ribosomal peptide synthase (NRPS, 26 hits), siderophore (20 hits), terpene (18 hits) and betalactone (13 hits). Additionally, PBGCs encoding resorcinol, redox-cofactors, type 3 and type 1 polyketide synthases (PKSs), non-alpha poly-amino acids (NAPAAs), linear azol(in)e-containing peptides (LAPs), ectoine, butyrolactone and aryl polyene were also found. Proportionally, the *Rhodococcus* strain APC 3903 had the greatest number of predicted secondary metabolite gene clusters at 28 (18% of the total). The presence of terpene BGCs is an indication that the orange colour observed in several of the colonies (Figure 4)., for example, the *Planococcus* spp. is the result of terpene/carotenoid productionAs with the BAGEL4 findings (Table 2), many of the predicted RiPP-like hits lacked an identifiable core biosynthetic gene, in which case the PBGC prediction may have been based on the presence of bacteriocin or RiPP-associated motifs or genes; this is indicated in where either no annotation was provided, or no known cluster hit was identified (Table 3).

The most promising candidates for further analysis, based on the presence of an identified core peptide/biosynthetic gene, were the putative cyclic bacteriocin in *Arthrobacter* APC 3897, the *Planococcus* spp. cerecidin-like lanthipeptides and the lactococcin 972-like peptides of *Curtobacterium* sp. APC 4022.

#### 2.4.1. Class I LanM-Group Bacteriocins (Lanthipeptides)

**Figure 8:**
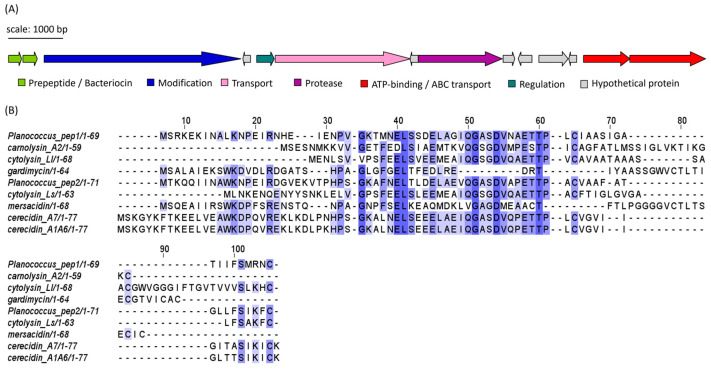
(**A**) Putative lanthipeptide (subclass II) gene cluster identified in *Planococcus* spp. isolates (*Planococcus* peptides 1 and 2). Identical clusters were found in APC 3900, APC 4015 and APC 4017. Genes are coloured according to predicted function. (**B**) MSA of the predicted *Planococcus* peptide precursor/bacteriocin sequence with most similar bacteriocin sequences identified using RiPPMiner: cytolysin Ll (*E. faecalis*), carnolysin A2 (*Carnobacterium maltaromaticum*), mersacidin (*B. amyloliquefaciens*), cytolysin Ls (*E. faecalis*), cerecidin A1-6 and cerecidin A7 (*Bacillus cereus*), gardimycin (*Actinoplanes liguriae*). The MSA is coloured according to shared identity of the aligned amino acid residues at a given position, with the colour shade deepening as the percentage of sequences sharing the same residue increases. No colour indicates <40% of sequences share the residue.

Isolates APC 3900, 4015 and 4016 of the genus *Planococcus* all encoded identical sequences, which corresponded to a cerecidin-like lanthipeptide (Figure 8A). The gene cluster encoded two premature/pro-peptides, a large modification protein (LanM-like), a transporter, a regulation protein, a protease and ATP-binding/ABC transport proteins. Several small open reading frames (sORFs) were also identified, but their function could not be determined. The two peptide precursors, denoted Pep1 and Pep2 for clarity, shared 52.94% identity with each other. When queried with RiPPMiner’s sequence similarity search tool, the top hits were cerecidin A1-6 and cerecidin A7 (*B. cereus*). Pep1 shared 46.27% and 44.78% identity with cerA7 and cerA1-6, respectively. Pep2 shared 54.29% and 55.71% id to cerA7 and cerA1-6, respectively. Other similar peptides from the database were cytolysin Ll (*E. faecalis*), carnolysin A2 (*Carnobacterium maltaromaticum*), gardimycin (*Actinoplanes liguriae*), mersacidin (*B. amyloliquefaciens*) and cytolysin Ls (*E. faecalis*). The multiple sequence alignment (MSA) of the *Planococcus* and RiPP hit sequences in Figure 8B shows only a few conserved residues within the region between residues 50 and 60. As with cerecidin, the Pep1 and Pep2 peptide sequences appear to possess a leader sequence, denoted by the GG/GA motif, followed by six additional amino acids which precede a potential core peptide (initiated by TT) [27]. The putative *Planococcus* modification gene shared 45.49% identity to LanM of cerecidin (Genbank accession: AHJ59542.1), indicating that the *Planococcus* peptides are class II lanthipeptides [28]. The statistics generated using EMBOSS Needle of the pairwise alignments of the precursor peptides, and the reference peptides are given in Table S3. Figures of the pairwise alignments are given in Figures S1 and S2.

#### 2.4.2. Class IIc (Circular) Bacteriocin

**Figure 9:**
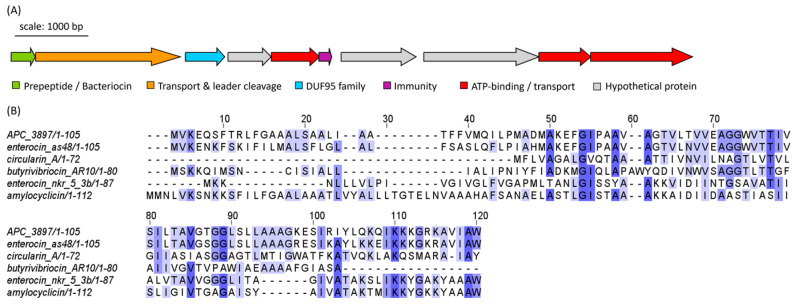
(**A**) Biosynthetic gene cluster (BGC) of a putative class IIc bacteriocin from *Arthrobacter* sp. isolate APC 3897. Genes are coloured according to predicted function. (**B**) Multiple sequence alignment (MSA) of APC 3897 BGC with similar bacteriocin sequences identified using RiPPMiner: butyrivibriocin (*Butyrivibrio fibrisolvens*), circularin A (*Clostridium beijerinckii*), enterocin NKR-5-3b (*Enterococcus faecium*), enterocin AS-48 (*E. faecalis*) and amylocyclicin (*Bacillus amyloliquefaciens*). The MSA is coloured according to shared identity of the aligned amino acid residues at a given position, with the colour shade deepening as the percentage of sequences sharing the same residue increases. No colour indicates <40% of sequences share the residue.

The cyclic bacteriocin gene cluster predicted in *Arthrobacter* isolate APC 3897 was annotated using BLASTp. The gene cluster consists of ten genes: a peptide precursor, a transporter/cleavage protein, a DUF95 family protein, three ATP-binding/transport proteins, an immunity protein and three hypothetical proteins of unknown function (Figure 9A). The predicted core precursor peptide sequence was queried using RiPPMiner: The closest related sequence was enterocin AS-48, a circular bacteriocin from *Enterococcus faecalis*, with which the peptide precursor sequences shared 71.3% identity. The other top bacteriocin/RiPP hits were butyrivibriocin (30.56%ID; *Butyrivibrio fibrisolvens*), circularin A (25.37%; *Clostridium beijerinckii*), enterocin NKR-5-3b (32.47%; *Enterococcus faecium*) and amylocyclicin (28.32%; *Bacillus amyloliquefaciens*). The peptide sequences were aligned and are illustrated in Figure 9B, which is coloured according to shared identity of the aligned amino acid residues and sequences are arranged according to pairwise identity. The pairwise alignment statistics and figures generated using EMBOSS Needle are provided in Table S3 and Figure S3, respectively.

#### 2.4.3. Class IId Bacteriocins

**Figure 10:**
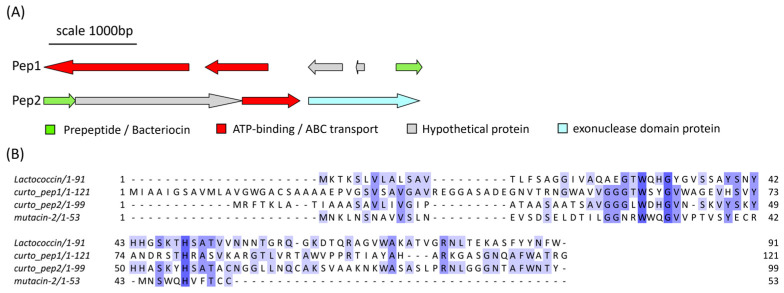
(**A**) The predicted gene clusters of the two lactococcin 972-like class IId bacteriocins from *Curtobacterium* sp. APC 4022, labelled pep1 and pep2. (**B**) The MSA of the peptide precursor sequences with mutacin II (*Streptococcus mutans*) and lactococcin 972 (*L. lactis* subsp. *lactis*) shows some conserved residues between the sequences The MSA is coloured according to shared identity of the aligned amino acid residues at a given position, with the colour shade deepening as the percentage of sequences sharing the same residue increases. No colour indicates <40% of sequences share the residue.

*Curtobacterium* sp. APC 4022 was found to contain two PBGCs located on two separate contigs that each encoded a lactococcin 972-like peptide (Figure 10A). The predicted gene cluster of the first peptide, denoted Pep1, consists of a core peptide gene, two ATP-binding/ABC-transport genes and two hypothetical genes of unknown function. The second gene cluster consists of four genes: a core peptide (Pep2) gene, a hypothetical gene of unknown function, an ATP-binding/ABC-transporter gene and a gene encoding an exonuclease family protein. When queried with RiPPMiner’s sequence similarity tool, the most similar hit to both core peptide sequences was mutacin II (*Streptococcus mutans*). The two *Curtobacterium* peptide sequences were aligned with mutacin II and lactococcin 972 (Figure 10B). Pep1 and Pep2 share 20.97% identity with each other. Pep1 shared 15.32% and 12.4% identity with lactococcin 972 and mutacin II, respectively. Pep2 shared 26% and 15% identity with lactococcin 972 and mutacin II, respectively. The pairwise alignment statistics generated using EMBOSS Needle are provided in Table S3. Figures of the pairwise alignments of *Curtobacterium* precursor peptides with the reference sequences are given in Figures S4 and S5. The pairwise alignment of Pep1 and Pep2 is also shown in Figure S4.

### 2.5. Antimicrobial Resistance Genes

The 36 deep-sea genomes were screened for AMR genes using ABRicate. Hits to resistance genes were only detected in the genome of *Rhodococcus* sp. APC 4022 (Table 4). Two genes corresponding to rifamycin/rifampin resistance were detected: (rif)iri, which encodes a rifampin (inactivating) monooxygenase and rpoB2, which encodes a resistant variant of the beta-subunit of RNA polymerase. A gene encoding a multidrug efflux protein (mtrA) was also detected.

## 3. Discussion

This study aimed to determine the bacteriocinogenic potential of bacterial isolates from the microbiomes of deep-sea fish. A combined approach of in vitro and in silico methods was used to identify antimicrobial-producing isolates and to further establish the potential source of this antimicrobial activity, with a focus on finding bacteriocin gene clusters.

The initial repeated screening against *L. bulgaricus* demonstrated that 44 of these isolates retained their bioactive activity (Table 1). The loss of activity by the remaining 21 isolates could be explained by the fact that antimicrobial production is often a tightly regulated process, dependent on the induction by external factors, such as other microbial metabolites present in the natural environment. Purification and removal of these isolates from the microbiome community may have affected their activity. Analysis of the spectrum of activity of these deep-sea isolates revealed that most strains had a narrow spectrum of activity. The only exceptions were *Pseudoalteromonas* strains APC 4017 and APC 3893 which were able to inhibit nine and eight of the indicator strains, respectively, with APC 4017 being particularly active against *E. faecium* 25644 and *Arthrobacter* sp. APC 3897 (Figure 6). Overall, just over half (11 out of 20) of the indicator strains were inhibited under the conditions tested. None of the Gram-negative indicator strains were impacted. Some inhibition patterns were noted amongst the taxonomic groups. The *Planococcus* strains were most effective against the *Planococcus* indicator strain APC 3906 and against the Micrococcales strains: *Arthrobacter* sp. APC 3897 and *Microbacterium* sp. APC 3901. The Gram-negative *Pseudoalteromonas* and *Psychrobacter* strains were only active against *L. bulgaricus* (with the aforementioned exceptions). The *Arthrobacter* strain APC 3897 was almost exclusively active against the *Listeria* strains. Based on the in vitro screening and taxonomic results, a number of isolates were chosen for whole-genome sequencing and in silico screening.

Interestingly, the whole-genome sequence data from the isolates was not found within the MAGs. This is a reflection of the difference in sample sizes between the two studies but also indicates that the isolates in this study may only represent a very small sample of the deep-sea fish microbiome, many of which may, in fact, be unculturable.

Through in silico screening using BAGEL4, we identified 19 AOIs, including lanthipeptide PBGCs and a cyclic bacteriocin PBGC. Though eleven of these AOIs lacked an identifiable core biosynthetic bacteriocin gene, the predictions by BAGEL4 may be based on similarities of the AOIs with bacteriocin-associated proteins. For example, in four of the predicted sactipeptide clusters, a gene was identified which encoded a putative GTP 3’,8-cyclase that shared similarity with MoaA, a member of the radical S-adenosylmethionine (rSAM) enzyme superfamily [29]. Specific rSAM enzymes (sactisynthases) are essential for forming the characteristic sactionine linkages of sactipeptides [30]. It is also possible that the core peptide genes are located elsewhere on these genomes, especially given that the in silico screening was carried out on contigs rather than full genomes. The lack of identifiable core peptides could also be due to the presence of unique motifs that are not found in the database.

The *Planococcus* strains APC 4015 and APC 4016, as mentioned above, demonstrated potent antimicrobial activity against several of the Gram-positive indicator strains, particularly against *Planococcus* sp. APC 3906, *Microbacterium* sp. APC 3901 and *Arthrobacter* sp. APC 3897. The *Planococcus* genomes were subsequently found to have identical gene clusters that encoded two putative bacteriocins. Of the known bacteriocin/RiPP sequences, the *Planococcus* peptides were most similar to the cerecidin peptides of *B.cereus*. Cerecidins are class II lanthipeptides that occur as two variants, CerA1 (of which there are six identical encoding genes, CerA1-6) and CerA7 [27]. The sequences also shared some similarities with the haemolytic enterotoxin of *E. faecalis*, cytolysin, which is composed of two structural subunits, CylL_L_ and CylL_s_ [31], and the two-component lantibiotic carnolysin (*C. maltaromaticum*) [32]. Cerecidin and cytolysin both contain two cleavage sites. The first occurs at a GA motif site after the first leader sequence. The second cleavage site occurs after a secondary leader sequence of six amino acids, leaving behind the core peptide [27,31]. These processing sites are also potentially present in the *Planococcus* peptides, as indicated by the area of homology seen in the region between residues 50 and 60 (Figure 8B). This suggests that these peptides undergo multistep processing.

*Arthrobacter* isolate APC 3897, which was observed to have anti-listerial activity in vitro, was found to contain genes encoding a class IIc cyclic bacteriocin. The predicted bacteriocin peptide precursor was found to be most closely related to enterocin AS-48, a circular bacteriocin from *Enterococcus faecalis*. AS-48 is most active against Gram-positive bacteria [33], particularly *Listeria* species [34], which is consistent with the in vitro activity we observed for *Arthrobacter* sp. APC 3897. The structure of the PBGC was also consistent with that of enterocin AS-48, each consisting of ten genes, including a structural peptide precursor, a transporter protein, ATP-binding/transport proteins and an immunity protein gene [35]. The *Arthrobacter* gene cluster was also found to encode a putative DUF95 family protein which is characteristic of circular bacteriocin gene clusters and is believed to be involved in the transport and secretion of these peptides [36]. To date, the identification of an AS-48-like circular bacteriocin has not been reported in an *Arthrobacter* strain; in fact, some *Arthrobacter* strains have been used as sensitive indicators in studies of AS-48 activity [37,38].

*Curtobacterium* sp. APC 4022 displayed very little in vitro antimicrobial activity, having only shown inhibition of *L. bulgaricus* when cultured on mTSA (Table 1). The genome of this strain was found to contain two separate gene clusters encoding predicted bacteriocins with sequence similarities, albeit low, to the class IId bacteriocins lactococcin 972 and mutacin II. Lactococcin 972, first isolated from *Lactococcus lactis* subsp. *lactis* IPLA972, inhibits peptidoglycan synthesis by targeting lipid II and is almost exclusively active against lactococci [39]. Members of the lactococcin 972 protein family (Pfam IPR006540) are usually associated with transmembrane putative immunity proteins. Mutacin II, from *S. mutans* NTCC 10449, is active against a range of Gram-positive species, including streptococci, and targets cell membrane functions [40].

The observed in vitro antimicrobial activity of the *Planococcus*, *Arthrobacter* and *Curtobacterium* strains may be attributed to their respective putatively identified bacteriocins; however, we cannot rule out the possibility that these strains also are producing other bioactive secondary metabolites. The production of bacteriocins can also be a tightly regulated process, which we expect holds true for these deep-sea strains. For example, the cerecidins of *B. cereus* are predicted to be under the control of competence quorum-sensing system genes (*comQXPA*), as well as the CerR regulatory protein, the stimulus of which is still unknown. Production of cerecidin in its native host has only been possible by constitutive expression of *cerR* using plasmids [41]. Further genomic and molecular analysis is needed to understand the regulatory systems behind the predicted bacteriocins of this study.

Lastly, we screened all of the genomes for the presence of AMR genes using ABRicate. Only three AMR genes were detected, and all three were from a single genome, that of *Rhodococcus* sp. APC 4022. Firstly, a resistant variant of the RNA polymerase beta subunit (RpoB2) was detected. Mutations to the RNA polymerase beta subunit (RpoB) can result in a gene duplication whereby one gene produces the rifamycin-sensitive RpoB and the second produces the resistant variant [42]. Possessing *rpoB* paralogs can be indicative of secondary metabolism and antibiotic production in Actinobacteria, though in vitro antimicrobial production was not detected in APC 4022 [43]. Secondly, a (*rif*)*iri* gene, which encodes a rifampin-inactivating monooxygenase, was identified. Production of rifamycin-inactivating or -modifying enzymes and mutations of RpoB are common resistance mechanisms and have previously been reported in *Rhodococcus* strains and related taxa; they are particularly well-studied in pathogenic bacteria such as *Mycobacteria* [44,45]. The third AMR gene detected was a multidrug efflux pump gene. The identification of multiple AMR genes indicates a potential multiple resistance mechanism utilised by *Rhodococcus* sp. APC 4022. The overall relative lack of detectable AMR genes across all the genomes is consistent with other reports of the low incidence of AMR genes in deep-sea fish-associated microbial genomes [22,46]. AMR genes have been detected in deep-sea water and sediment, but the incidence may be low compared to sample sites heavily impacted by anthropogenic activities [47,48]. There have been suggestions that AMR genes found in marine environments are largely unclassified [49]; therefore, the accurate identification of AMR genes in deep-sea samples may be even more challenging.

It is particularly notable that we were able to successfully isolate strains capable of targeting food-borne pathogens. Foodborne disease is a huge threat to public health and safety. The World Health Organisation (WHO) has estimated that globally up to 1 in 10 people fall ill to foodborne disease every year, leading to almost half a million premature deaths and huge economic losses [50]. It is important, now more than ever, to be able to find novel compounds for combatting foodborne pathogens, particularly when treatments are being compounded by the increasing incidence of antimicrobial resistance. The aquaculture industry is another potential application of deep-sea bacteriocins, which we have not explored here in detail. For example, the overuse of antibiotics and the development of antimicrobial resistance is also a great concern in the fish farming industry, with disease outbreaks having significant economic and health impacts [51].

## 4. Materials and Methods

### 4.1. Isolation and Cultivation of Antimicrobial-Producing Isolates

**Figure 11:**
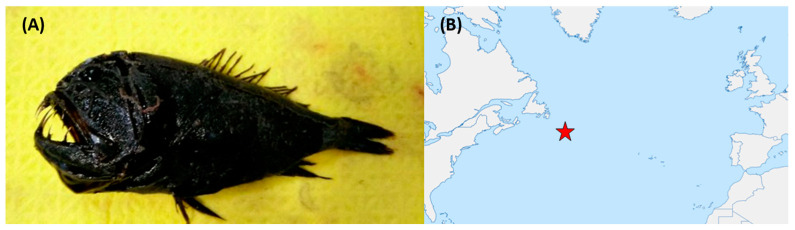
(**A**) *Anoplogaster cornuta*—an example of one of the fish species from which bioactive bacteria were isolated. (**B**) Approximate sampling location (indicated by the red star icon) in the Atlantic Ocean. Deep-sea fish specimens were collected from depths of approximately 1000 m.

Fish were collected from sample sites in international waters off the Grand Banks of Newfoundland at depths ranging from 850 m to 1000 m, as previously described [22]. An example of one the fish specimens and the sampling location is shown in Figure 11. Swabs were taken from the skin and intestines of the fish, plated onto BD Difco^TM^ Marine Agar 2216 (MA, Becton Drive, NJ, USA) and incubated at 4 °C for three weeks. Individual colonies (n = 374) were picked and stocked in 35% glycerol and stored at −80 °C.

### 4.2. In Vitro Screening

Previous work in our laboratory (not published) conducted preliminary screening of a cohort of 374 stocked isolates against the indicator strain *Lactobacillus delbrueckii* subsp. *bulgaricus* LMG 6901 and identified 65 strains with antimicrobial activity. In this study, we reassessed the activity against *L. bulgaricus* by the 65 strains and examined the spectrum of antimicrobial activity of these strains against various target bacteria strains (listed in Table 5).

For analysis of activity against *L. bulgaricus*, isolates were plated on MA, brain heart infusion (BHI, Oxoid, Basingstoke, UK) agar and modified tryptic soy agar (mTSA, +0.5 g/L NaCl, Oxoid) and incubated at 5 °C (±1 °C) for two weeks. The plates were then overlaid with sloppy media containing 0.8% agar and 0.25% of an overnight culture of *L. bulgaricus* in modified Difco MRS (mMRS, +0.5 g/L cysteine). Following incubation overnight, we measured the distance from the edge of the producing colony to the edge of the area of growth of the indicator organism and recorded the results qualitatively as follows: + (0.5–2.5 mm), ++ (2.6–5 mm), +++ (5.1–10 mm) and ++++ (>10 mm).

For subsequent colony overlay screening to assess the spectrum of activity, isolates were plated on MA and incubated at 5 °C (±1 °C) for 2 weeks. The plates were then overlaid with indicator organisms in the relevant sloppy media, as listed in Table 5, using the same method as mentioned above. Inhibition distances were measured after overnight incubation or after 48 h for indicators cultured at 20 °C.

### 4.3. 16S rRNA Gene Sequencing and Taxonomy

Colony PCR was performed on all of the isolates. Cells were lysed by resuspending in 10 µL lysis buffer (20 mM Tris-HCL, pH 8.0, 2 mM EDTA, 1.2% Triton X-100; reagents from Sigma-Aldrich/Merck, Burlington, Massachusetts, USA) and incubatingat 95 °C for 10 min. PCR was performed in a total volume of 50 μL using 25 μL of BioMix^TM^ Red (Meridian Bioscience^®^, London, UK), 16 μL of sterile molecular-grade water, 2 μL (at 10 pmol/µL) of the non-specific universal primers 27F (5′-AGAGTTTGATCCTGGCTCAG-3′) and 1492R (5’-GGTTACCTTGTTACGACTT-3’) and 5 μL of DNA template from lysed cells. The following amplification conditions were used: 95 °C for 1 min, followed by 32 cycles of 95 °C for 15 s, 55 °C for 15 s and 72 °C for 32 s with a final extension at 72 °C for 10 min. PCR products were run on a 1.5% agarose gel, stained with MIDORI Green Advance and subject to gel electrophoresis. The gel was visualised using a UV-transilluminator to confirm the presence of amplified DNA. PCR products were purified using the GeneJET PCR purification Kit (Thermo Scientific, Waltham, MA, USA). DNA sequencing of both the forward and reverse strands was performed by GENEWIZ (Azenta Life Sciences, Leipzig, Germany) or Source BioScience (Waterford, Ireland). Where possible, sequence pairs were concatenated before analysis. Where available, 16S rRNA gene sequences were also extracted from subsequent gDNA sequencing data and used for inference. Taxonomy was inferred by searching the resulting 16S rRNA gene sequences for sequence similarity with NCBI’s 16S rRNA database using the BLAST tool (https://blast.ncbi.nlm.nih.gov/Blast.cgi, accessed on 3 November 2021).

### 4.4. Genomic DNA Extraction and Sequencing

Genomic DNA (gDNA) was extracted from 36 of the isolates using the GeneJET Genomic DNA Purification Kit (Thermo Scientific) and the relevant protocol, Gram-negative or Gram-positive, for the isolate. DNA quality was checked using agarose (1%) gel electrophoresis. An overview of the sequencing and assembly information of each sample is given in Table S1. In brief, the first set of gDNA samples were sequenced on an Illumina MiSeq platform (2 × 250 bp paired-end reads) at GenProbio s.r.l. (Parma, Italy). The second set of samples were sequenced on the Illumina MiSeq platform (2 × 250 bp paired-end reads) by MicrobesNG (http://www.microbesng.com), which is supported by the BBSRC, (grant number BB/L024209/1). APC 4015 was sequenced by GENEWIZ (Azenta Life Sciences, Burlington, MA, USA) on the Illumina NovaSeq platform (2 × 150 bp paired-end reads).

### 4.5. Genome Assembly and Annotation

Reads provided by MicrobesNG were processed and assembled by the sequencing company. For all other samples, read quality was assessed with FastQC (v0.11.9; https://www.bioinformatics.babraham.ac.uk/projects/fastqc), trimmed with Trimmomatic (v0.36) [52] to a Phred score of 20 across a 4 bp sliding window. Reads less than 50 bp were discarded. Contigs (>500 bp) were assembled using SPAdes (v3.15.3) [53], and assembly quality was assessed using QUAST (v4.4) [54] and CheckM (v1.0.18) [55]. Assemblies were submitted to Genbank and annotated using the NCBI Prokaryotic Annotation Pipeline (PGAP) upon submission [56]. An overview of the assembly information, QUAST and CheckM statistics is given in Table S1. Additionally, the 16S rRNA gene sequences from the WGS data were searched against the SILVA rRNA gene project database [57]. The resulting taxonomic descriptions and closest related reference strain(s) are reported in Table S2.

The datasets generated in this study are available under the BioProject accession number PRJNA883941.

### 4.6. In Silico Antimicrobial Screening

Assembled genomes were screened for putative bacteriocin and secondary metabolite gene clusters using the genome mining tools BAGEL4 (BAGEL v4) [26] and AntiSMASH (v.5.1.2.) [58], respectively.

### 4.7. Putative Biosynthetic Gene Cluster (PBGC) Analysis

Putative core precursor peptide genes were analysed using RiPPMiner’s sequence similarity search [59] to query their similarity to known RiPPs from the database. Multiple alignments of the core peptide sequences and the RiPPMiner hits were made using Clustal Omega [60] and visualised using Jalview [61]. Pairwise alignments between the precursor peptide sequences and the RiPPMiner hits/reference sequences were generated using EMBOSS Needle [62] using default parameters. The matrix used for each pairwise alignment is provided along with the results in Table S3. Genes within the PBGCs adjacent to the core peptide genes were queried and annotated using BLASTp to determine their potential function. The annotated PBGCs were visualised using Gene Graphics [63].

### 4.8. Antimicrobial Resistance Gene Screening

Genomes were screened for the presence of antimicrobial resistance genes using ABRicate (https://github.com/tseemann/abricate). The databases used were ARG-ANNOT [64], CARD [65], EcOH [66], MEGARES [67], NCBI [68], PlasmidFinder [69], Resfinder [70] and VFDb [71].

## 5. Conclusions

This study has shown that not only are bacteria from the skin and gut microbiomes of deep-sea fish cultivable, but they are a potentially rich source of antimicrobial agents. The majority of the antimicrobial producers in this study were Gram-negative *Pseudoalteromonas* strains of the phylum Pseudomonadota, though some of the most potent producers included *Planococcus* and *Arthrobacter* strains of the phyla Bacillota and Actinomycetota, respectively. Whilst initially selected based on their inhibitory activity against *Lactobacillus bulgaricus*, it was demonstrated that these deep-sea isolates could inhibit foodborne pathogens, such as *Listeria monocytogenes* and *Enterococcus* spp. Overall, the most susceptible indicators were other native marine strains, or more closely related organisms, a characteristic of the narrow spectrum of activity of many bacteriocins. In silico screening found that these isolates encode a wide variety of PBGCs encoding antimicrobial molecules from type I and II bacteriocins to LAPs and sactipeptides as well as PKSs, NRPSs, betalactones and terpenes. Included in these are potentially novel bacteriocins and variants of known bacteriocins. This provides great scope for future work in exploring applications of targeting pathogenic bacteria with antimicrobials that are produced, and potentially active at low temperatures. The dual in vitro and in silico approach allowed for a more comprehensive understanding of the potential targets of deep-sea bacteriocins and an insight into the antimicrobial molecules that they may produce.

## Figures and Tables

**Table 1:** Results of inhibitory activity observed against the target organism *Lactobacillus delbrueckii* subsp. *bulgaricus (L. bulgaricus)* on selected media. The isolate sample source and fish host are also given and numbered as follows: 1, *Anoplogaster cornuta*; 2, A*pristurus* sp.; 3, *Bathysaurus ferox*; 4, *Centroscymnus coelolepis*; 5, *Malacosteus niger*; 6, *Notacanthus chemnitzii*; and 7, *Alepocephlus bairdii*. Inhibition is indicated by + (0.5–2.5 mm), ++ (2.6–5 mm), +++ (5.1–10 mm), or − (no inhibition).

	Activity vs. *L. bulgaricus LMG 6901*			
Isolate	MA	BHI	mTSA	Source (Sample, Host)
*Arthrobacter* sp. APC 3897	−	+	+	skin, 6
*Curtobacterium* sp. APC 4022	−	−	+	intestine, 4
*Photobacterium* sp. *APC 3280*	−	+	+	intestine, 4
*Planococcus* sp. 26D.a_F	−	nd	+++	skin, 6
*Pseudoalteromonas* spp.				
APC 3495	+	+	+	intestine, 1
APC 3213	+	+	++	intestine, 2
APC 3238	+	+	+	intestine, 3
APC 3419	+	ng	+	intestine, 3
APC 3695	−	+	+++	intestine, 3
APC 4023	+	+	+	intestine, 3
APC 4024	+	+	+++	intestine, 3
APC 3284	+	ng	ng	intestine, 4
APC 3355	++	+++	ng	intestine, 4
APC 3356	++	nd	−	intestine, 4
APC 3358	++	+++	++	intestine, 4
APC 3412	++	ng	ng	intestine, 5
APC 3224	+	ng	ng	intestine, 6
APC 3391	+	++	+	skin, 7
APC 3502	+	ng	+	skin, 1
APC 4025	+	+	+	skin, 2
APC 4026	+	+	+	skin, 2
APC 3893	++	ng	ng	skin, 3
APC 3895	+	ng	−	skin, 3
APC 3896	−	+++	+	skin, 3
APC 4017	+	ng	ng	skin, 3
APC 3274	−	+++	+	skin, 4
APC 3691	+	ng	ng	skin, 4
APC 3407	+	+	+	skin, 5
APC 3904	−	+	+	skin, 5
APC 3221	++	ng	+	skin, 6
APC 3250	+	+	+	skin, 6
APC 4019	+	nd	+	skin, 6
APC 4020	+	nd	++	skin, 6
*Psychrobacter* spp.				
5A.1	−	++	+	skin, 4
5A.2	−	+++	+	skin, 4
APC 3272	−	++	−	skin, 6
APC 3275	−	+	+	intestine, 6
APC 3276	−	+	+	skin, 4
APC 3277	+	+	+	skin, 4
APC 3350	+	++	−	skin, 4
APC 3426	−	++	+++	skin, 3
APC 3692	++	nd	+	skin, 6
APC 4028	−	++	+	skin, 4

MA = BD Difco 2216 marine agar; BHI = brain heart infusion; mTSA = tryptic soy agar +0.5 g/L NaCl; ng = no growth of producing strain; nd = no measurement data.

**Table 2:** Summary of the potential bacteriocin/RiPP gene clusters and their annotations predicted by BAGEL4 in the deep-sea bacterial genomes. The predicted bacteriocins included subclass II lanthipeptides (with similarity to mersacidin/cerecidin), subclass III lanthipeptides and a circularin A-like class II bacteriocin. Other predicted bacteriocin subtypes included sactipeptides, a LAP and a lactococcin 972-like peptide. Predicted AOIs which lacked an identifiable core peptide gene and could not be annotated by BAGEL4 are given as “undefined”. ×2 = two clusters found; %sim = % similarity.

Strain	BAGEL4		
	Prediction	Annotation	% Sim.
*Arthrobacter* sp. APC 3897	circularin_A	bacteriocin class cyclical uberolysin-like	67%
	sactipeptide	GTP 3’,8-cyclase (moaA)	73.70%
*Curtobacterium* sp. APC 4022	lactococcin_972	bacteriocin (Lactococcin_972)	39.70%
	sactipeptide	GTP 3’,8-cyclase (moaA)	59.50%
*Kocuria* sp. APC 4018	linocin-M18	encapsulating protein for peroxidase	58.90%
*Planococcus* sp. APC 3900	bacteriocin mersacidin/cerecidin (×2)	bacteriocin class II with double-glycine leader (×2)	50–55.6%
*Planococcus* sp. APC 4015	bacteriocin mersacidin/cerecidin (×2)	bacteriocin class II with double-glycine leader peptide (×2)	50–55.6%
	sactipeptide	undefined	-
*Planococcus* sp. APC 4016	bacteriocin mersacidin/cerecidin (×2)	bacteriocin class II with double-glycine leader (×2)	50–55.6%
*Pseudoalteromonas* sp. APC 3224	zoocin_A	undefined	36.40%
*Pseudoalteromonas* sp. APC 3426	sactipeptide	undefined	-
*Pseudoalteromonas* sp. APC 3893	lanthipeptide class III	undefined	-
*Pseudoalteromonas* sp. APC 4017	lanthipeptide class III	undefined	-
*Psychrobacter* sp. APC 3350	sactipeptide	probable GTP 3’,8-cyclase	-
*Rhodococcus* sp. APC 3903	lanthipeptide class III	undefined	-
	LAPs	undefined	-
	linocin_M18/putative bacteriocin family protein	encapsulating protein for peroxidase	66.70%
	sactipeptide	undefined	-
*Winogradskyella* sp. APC 3343	sactipeptide	GTP 3’,8-cyclase (moaA)	41.40%

**Table 3:** In silico predictions by AntiSMASH of bacteriocin/RiPP-like PBGCs and other secondary metabolite gene clusters. Where no core peptide or no similar cluster was found, the result is given as “none.” ×2 = two clusters found; % sim = similarity; nd = no data.

Strain	AntiSMASH			
	Bacteriocin Prediction	Similar Cluster	% Sim.	Other PBGCs Present
*Arthrobacter* sp. APC 3897	RiPP-like	head-to-tail cyclized peptide	71	betalactone (×2), siderophore, T3PKS, terpene
*Curtobacterium* sp. APC 4022	RiPP-like (×2)	lactococcin_972 (×2)	nd	betalactone, NRPS-like, siderophore, T3PKS (×2), terpene
*Kocuria* sp. APC 4018	RiPP-like	linocin_M18	nd	betalactone, NRPS-like, siderophore, T3PKS, terpene
*Microbacterium* sp. APC 3898	none	-	-	betalactone, NAPAA, T3PKS, terpene
*Microbacterium* sp. APC 3901	none	-	-	NAPPA, T3PKS, betalactone, terpene
*Micrococcus* sp. APC 4021	none	-	-	betalactone, ectoine, NRPS-like, siderophore, terpene
*Planococcus* sp. APC 3900	lanthipeptide class II	cerecidin	70	terpene (×2)
*Planococcus* sp. APC 3906	none	-	-	terpene (×2)
*Planococcus* sp. APC 4015	lanthipeptide	cerecidin	70	terpene (×2)
*Planococcus* sp. APC 4016	lanthipeptide class II	cerecidin	70	terpene (×2)
*Pseudoalteromonas* sp. APC 3213	RiPP-like	burkholderic acid	15	siderophore
*Pseudoalteromonas* sp. APC 3215	RiPP-like	none	-	siderophore
*Pseudoalteromonas* sp. APC 3218	RiPP-like	none (DUF692-family protein)	nd	siderophore
*Pseudoalteromonas* sp. APC 3224	RiPP-like (×2)	none (DUF692-family protein, ×2)	nd	NRPS, siderophore
*Pseudoalteromonas* sp. APC 3227	RiPP-like	none	-	siderophore
*Pseudoalteromonas* sp. APC 3250	RiPP-like	none	-	siderophore
*Pseudoalteromonas* sp. APC 3350	RiPP-like	none (DUF692-family protein)	nd	betalactone, redox-cofactor, siderophore
*Pseudoalteromonas* sp. APC 3356	RiPP-like	none	-	siderophore
*Pseudoalteromonas* sp. APC 3358	RiPP-like	nucleocidin	17	arylpolyene
*Pseudoalteromonas* sp. APC 3426	none	-	-	betalactone
*Pseudoalteromonas* sp. APC 3495	RiPP-like	none (DUF692-family protein)	nd	siderophore
*Pseudoalteromonas* sp. APC 3691	RiPP-like	none (DUF692-family protein)	nd	arylpolyene, betalactone
*Pseudoalteromonas* sp. APC 3694	RiPP-like	none (DUF692-family protein)	nd	none
*Pseudoalteromonas* sp. APC 3893	RiPP-like	none	-	arylpolyene, resorincol, NRPS (×2), PKS, T3PKS
*Pseudoalteromonas* sp. APC 3894	RiPP-like	none (DUF692-family protein)	nd	siderophore
*Pseudoalteromonas* sp. APC 3895	RiPP-like	none	-	siderophore
*Pseudoalteromonas* sp. APC 3896	RiPP-like	none	-	siderophore
*Pseudoalteromonas* sp. APC 3907	RiPP-like	none	-	siderophore, betalactone
*Pseudoalteromonas* sp. APC 4017	RiPP-like	none	-	arylpolyene, resorcinol (×2), PKS, NRPS, T3PKS
*Pseudoalteromonas* sp. APC 4026	RiPP-like	none (DUF692-family protein)	nd	siderophore
*Psychrobacter* sp. *5A.1*	RiPP-like	none (DUF692-family protein)	nd	betalactone, redox-cofactor, siderophore
*Psychrobacter* sp. APC 3279	none	-	-	betalactone
*Psychrobacter* sp. APC 3281	none	-	-	betalactone, redox-cofactor
*Psychrobacter* sp. APC 3355	RiPP-like (×2)	none (DUF692-family protein, ×2)	nd	siderophore
*Rhodococcus* sp. APC 3903	LAP	dissonitrile antibiotic SF2768	11	butyrolactone, ectoine, NRPS (×15), NRPS-like (×3), terpene (×2), PKS-like, T1PKS (×2),
	RiPP-like	branched-chain fatty acids	75	
	lanthipeptide class III	none	-	
*Winogradskyella* sp. APC 3343	none	-	-	NRPS, T1pks, T3PKS, Terpene (×2)

**Table 4:** The output of ABRicate genome screening for antimicrobial resistance (AMR) genes. All hits came from the genome sequence of *Rhodococcus* sp. APC 4022.

Contig	Gene	Resistance	%ID	Database	Product	Accession
6	*rpoB2*	rifamycin/rifampin	80	card	beta-subunit of RNA polymerase (RpoB2)	AP006618.1:4835199-4838688
8	(*rif*)*iri*	rifamycin/rifampin	97.81	argannot	(Rif)iri	U56415:280-1719
8	*iri*	rifamycin/rifampin	97.81	ncbi	rifampin monooxygenase Iri	NG_047911.1
8	*iri*	rifamycin/rifampin	97.81	card	rifampin monooxygenase Iri	U56415:279-1719
8	*iri*	rifamycin/rifampin	97.81	megares	rifampin monooxygenase Iri	MEG_3434
9	*mtrA*	macrolide;penam	80.41	card	transcriptional activator of multidrug efflux pump (MtrCDE)	AL123456.3:3627349-3626662
9	*mtrAD*	multidrug	80.41	megares	Multi-drug RND efflux regulator MTRAD	MEG_4078

**Table 5:** The target strains and respective growth conditions used in this study.

Species	Strain ID	Temperature	Atmosphere	Growth Media	Notes
*Arthrobacter* sp.	APC 3897	20 °C	aerobic	MA	
*Bacillus cereus*	DPC 6087	37 °C	aerobic	BHI	
*Enterococcus faecalis*	OG1RF	37 °C	aerobic	BHI	
*Enterococcus faecium*	DSM 25644	37 °C	aerobic	BHI	
*Escherichia coli*	MG1655	37 °C	aerobic	LB	
*Kocuria* sp.	APC 4018	20 °C	aerobic	MA	
*Lactobacillus acidophilus*	EM066-BC-T3-3	37 °C	anaerobic	MRS	+0.5 g/L cysteine
*Lactobacillus delbrueckii* subsp. *bulgaricus*	LMG 6901	37 °C	aerobic	MRS	+0.5 g/L cysteine
*Lactococcus lactis* subsp. *cremoris*	HP	37 °C	aerobic	GM17	
*Listeria innocua*	ATCC 33090	30 °C	aerobic	BHI	
*Listeria monocytogenes*	EDG-e	37 °C	aerobic	BHI	
*Microbacterium* sp.	APC 3901	20 °C	aerobic	MA	
*Micrococcus luteus*	DSM 1790	30 °C	aerobic	BHI	
*Planococcus* sp.	APC 3906	20 °C	aerobic	MA	
*Pseudomonas aeruginosa*	PA01	37 °C	aerobic	BHI	
*Psychrobacter* sp.	APC 3276	20 °C	aerobic	MA	
*Rhodococcus* sp.	APC 3903	20 °C	aerobic	MA	
*Salmonella enterica* ser. Typhimurium	DPC 6046	37 °C	aerobic	BHI	
*Staphylococcus aureus*	RN4220	37 °C	aerobic	BHI	
*Staphylococcus intermedius*	DSM 20373	37 °C	aerobic	BHI	
*Streptococcus pyogenes*	DPC 6992	37 °C	aerobic	BHI	
*Vibrio fischeri*	n/a	20 °C	aerobic	MA	

ATCC, American Type Culture Collection; APC, APC Microbiome Ireland Culture Collection; DSM, Leibniz Institute DSMZ-German Collection of Microorganisms and Cell Cultures; DPC, Teagasc Culture Collection; LMG, Belgian Coordinated Collections of Microorganisms (BCCM).

## Data Availability

The datasets generated in this study are available under the BioProject accession number PRJNA883941.

## Supplementary Materials

The following supporting information can be downloaded at: https://www.mdpi.com/article/10.3390/md21080444/s1; Table S1: Overview of the whole-genome sequencing data, QUAST and CheckM metrics; Table S2: Taxonomic descriptions, based on the 16S rRNA gene sequence, of the strains selected for whole-genome sequencing. Sequences were searched against the SILVA rRNA gene project database; Table S3: Resulting statistics from the pairwise alignments of precursor peptide sequences using EMBOSS Needle. Figure S1: Pairwise alignment of the *Planococcus* precursor peptide, Pep1, with (A) Pep2 (*Planococcus* spp.); (B) cerecidin A1A6 (*B. cereus*); (C) cerecidin A7 (*B. cereus*); (D) cytolysin Ls (*E. faecalis*); (E) carnolysin A2 (*C. maltaromaticum*); (F) carnolysin A1 (*C. maltaromaticum*); (G) cytolysin Ll (*E.faecalis*); (H) gardimycin (*A. liguriae*); (I) mersacidin (*B. amyloliquifaciens*). Figure S2: Pairwise alignment of the *Planococcus* precursor peptide, Pep2, with (A) cerecidin A7 (*B. cereus*); (B) cerecidin A1A6 (*B. cereus*); (C) cytolysin Ls (*E. faecalis*); (D) carnolysin A1 (*C. maltaromaticum*); (E) carnolysin A2 (*C. maltaromaticum*); (F) cytolysin Ll (*E.faecalis*); (G) gardimycin (*A. liguriae*); (H) mersacidin (*B. amyloliquifaciens*). Figure S3: Pairwise alignment of the *Arthrobacter* sp. APC 3897 precursor peptide with (A) enterocin AS48 (*E. faecalis*); (B) amylocyclicin (*B. amyloliquifaciens*); (C) circularin A (*C. beierinckii*); (D) enterocin nkr_5_3b (*E. faecium*); (E) butyrivibriocin AR10 (*B. fibriosolvens*). Figure S4: Pairwise alignment of the *Curtobacterium* sp. APC 4022 precursor peptide curto_Lct1 with (A) curto_Lct2); (B) lactococcin 972 (*L. lactis*); (C) mutacin 2 (*S. mutans*). Figure S5: Pairwise alignment of the *Curtobacterium* sp. APC 4022 precursor peptide curto_Lct2 with (A) lactococcin 972 (*L. lactis*) and (B) mutacin 2 (*S. mutans*).
