# Supplementary figures and images for: Bioactivity Screening and Genomic Analysis Reveals Deep-Sea Fish Microbiome Isolates as Sources of Novel Antimicrobials
Source: Mar Drugs. 2023 Aug 7;21(8):444. doi: 10.3390/md21080444 (PMC10456417; doi:10.3390/md21080444)

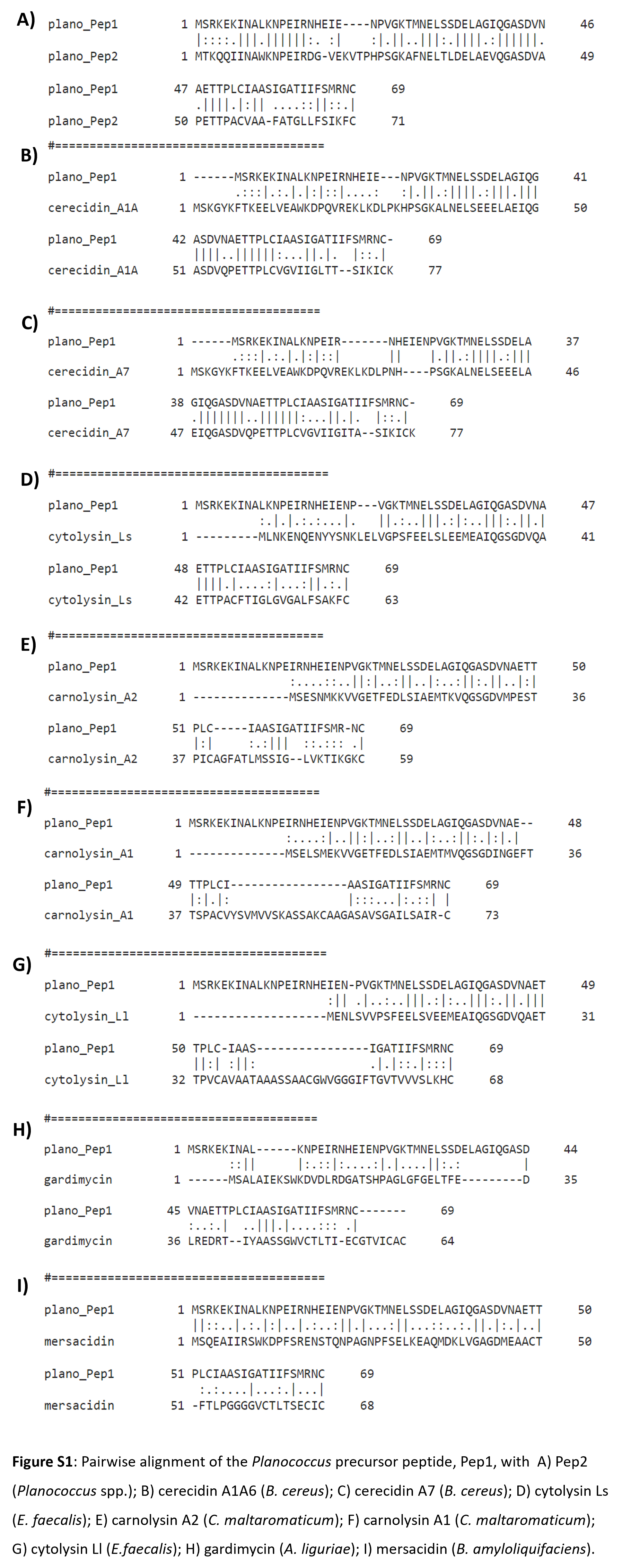

Supplement: Supplementary file 1 [file marinedrugs-21-00444-s001.zip › supplementary_material/Figure_S1.tif]

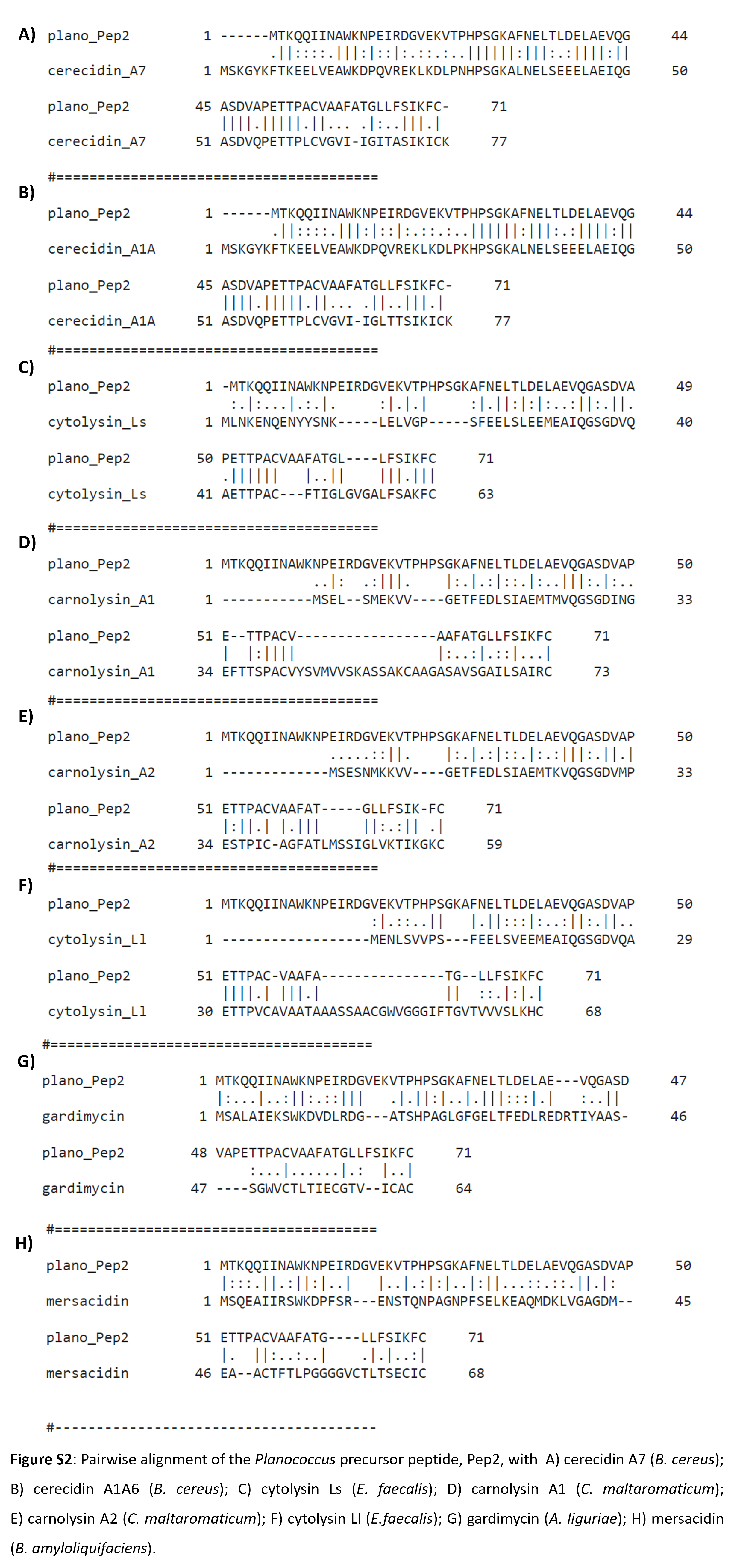

Supplement: Supplementary file 1 [file marinedrugs-21-00444-s001.zip › supplementary_material/Figure_S2.tif]

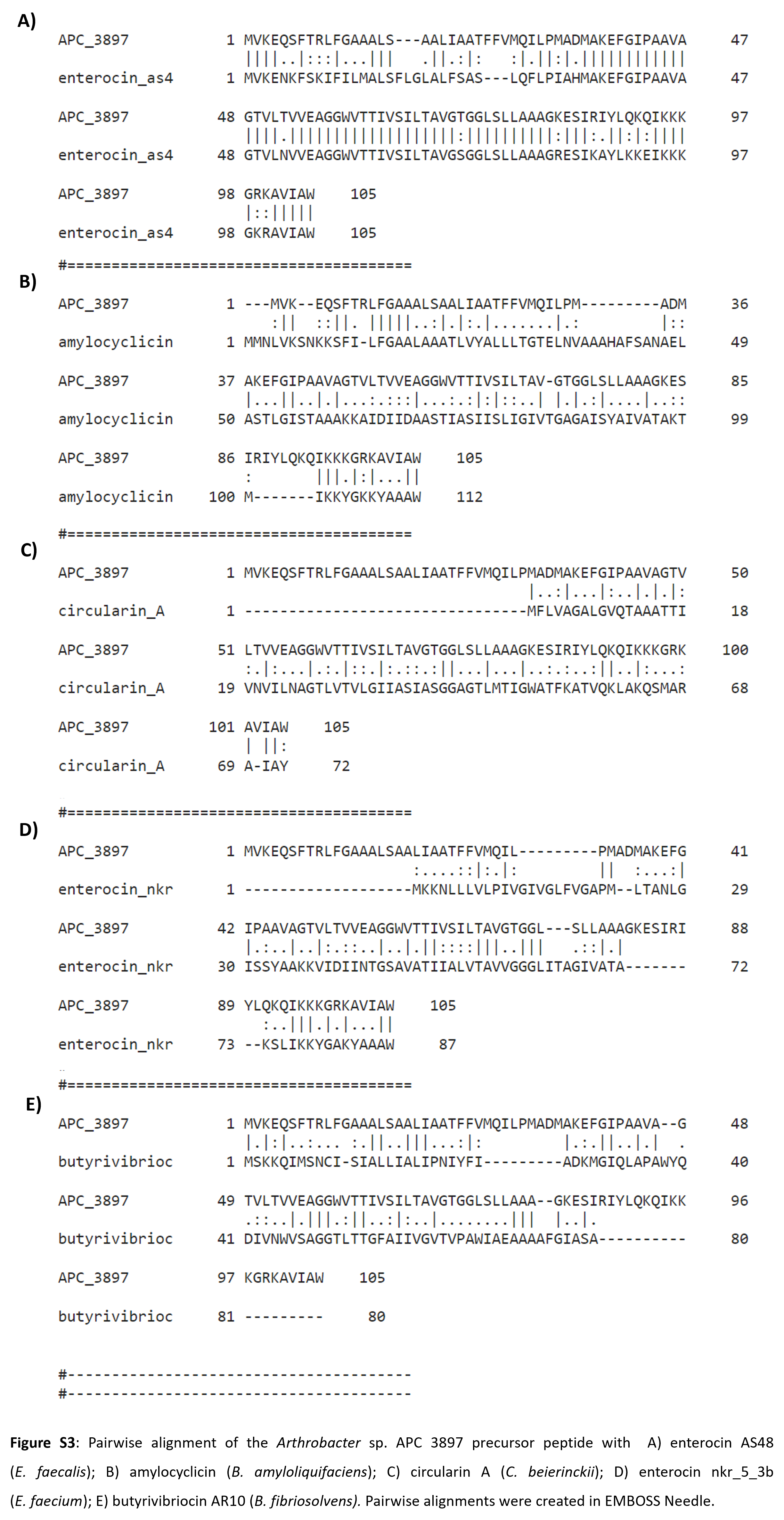

Supplement: Supplementary file 1 [file marinedrugs-21-00444-s001.zip › supplementary_material/Figure_S3.tif]

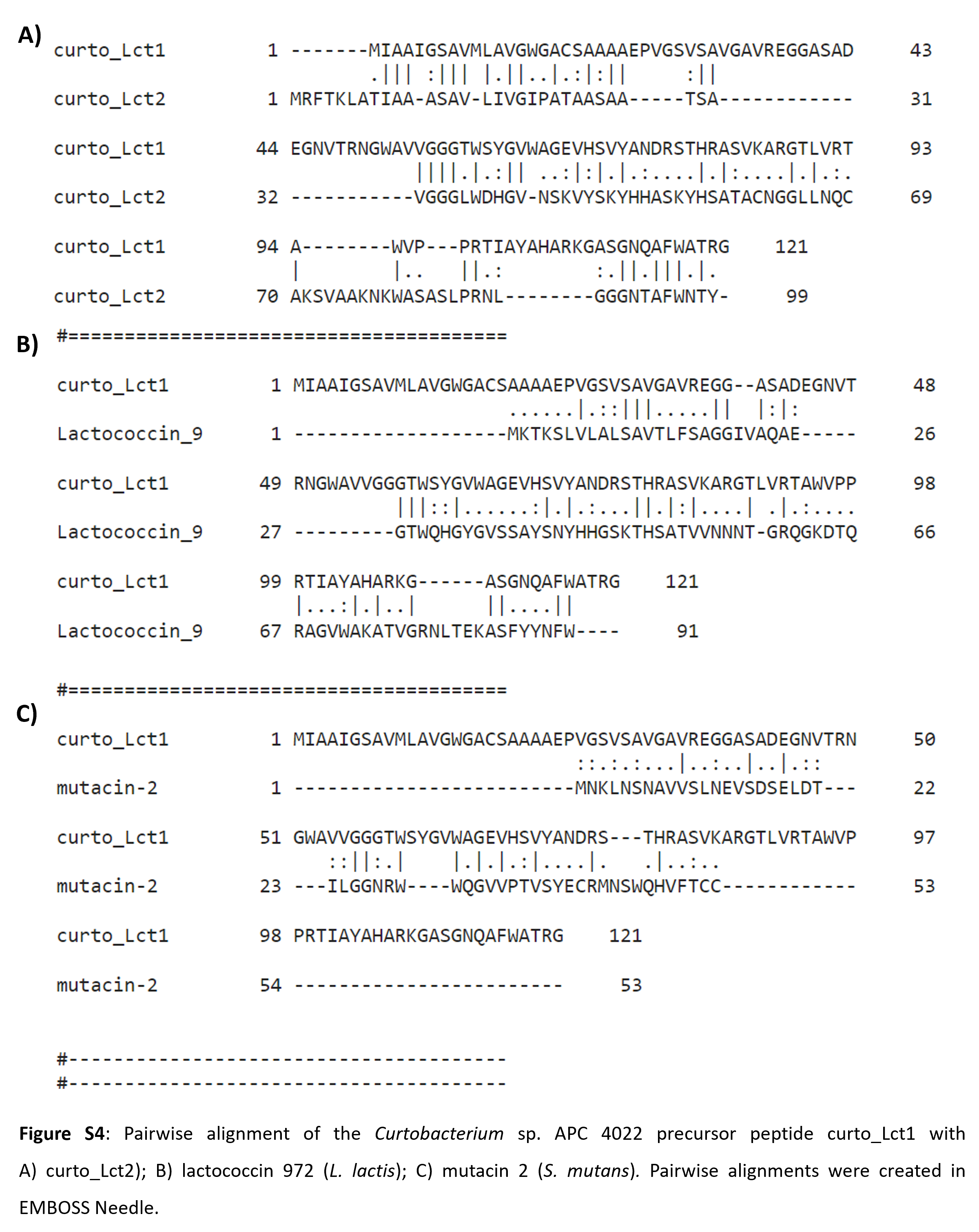

Supplement: Supplementary file 1 [file marinedrugs-21-00444-s001.zip › supplementary_material/Figure_S4.tif]

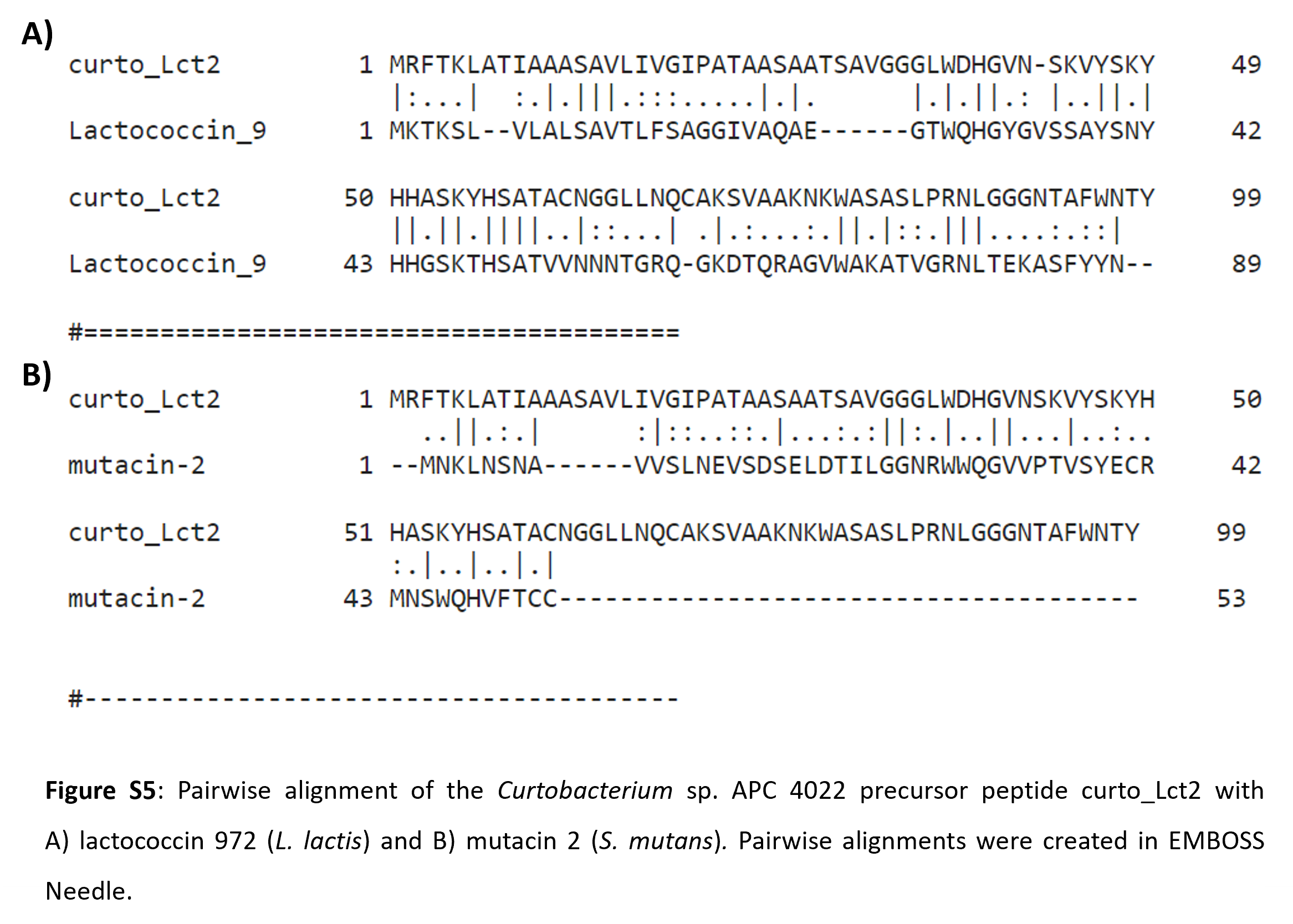

Supplement: Supplementary file 1 [file marinedrugs-21-00444-s001.zip › supplementary_material/Figure_S5.tif]
